# RU-SLAM: A Robust Deep-Learning Visual Simultaneous Localization and Mapping (SLAM) System for Weakly Textured Underwater Environments

**DOI:** 10.3390/s24061937

**Published:** 2024-03-18

**Authors:** Zhuo Wang, Qin Cheng, Xiaokai Mu

**Affiliations:** 1 Science and Technology on Underwater Vehicle Laboratory, Harbin Engineering University, Harbin 150001, China; 2 Qingdao Innovation and Development Center, Harbin Engineering University, Qingdao 266000, China

**Keywords:** autonomous underwater vehicle, underwater image, underwater SLAM, deep learning, local and global features

## Abstract

Accurate and robust simultaneous localization and mapping (SLAM) systems are crucial for autonomous underwater vehicles (AUVs) to perform missions in unknown environments. However, directly applying deep learning-based SLAM methods to underwater environments poses challenges due to weak textures, image degradation, and the inability to accurately annotate keypoints. In this paper, a robust deep-learning visual SLAM system is proposed. First, a feature generator named UWNet is designed to address weak texture and image degradation problems and extract more accurate keypoint features and their descriptors. Further, the idea of knowledge distillation is introduced based on an improved underwater imaging physical model to train the network in a self-supervised manner. Finally, UWNet is integrated into the ORB-SLAM3 to replace the traditional feature extractor. The extracted local and global features are respectively utilized in the feature tracking and closed-loop detection modules. Experimental results on public datasets and self-collected pool datasets verify that the proposed system maintains high accuracy and robustness in complex scenarios.

## 1. Introduction

In recent years, significant strides have been achieved in the research on Autonomous Underwater Vehicles (AUVs) with the increasing demand for marine resources. AUVs have emerged as a pivotal tool in the exploration and development of oceanic resources [1]. To undertake diverse missions effectively and safely, precise self-positioning is paramount for AUVs. The current dominant methods for AUV positioning encompass inertial navigation systems (INSs), acoustic positioning systems, geophysical navigation, and Simultaneous Localization and Mapping (SLAM) [2,3,4]. However, the accuracy of INSs gradually diminishes over time. Acoustic positioning systems and geophysical navigation suffer from pre-arranged transponders and pre-existing maps for localization, respectively. In contrast, SLAM enables AUVs to locate objects in unknown environments without prior information, which can reduce cumulative errors through back-end optimization and loop closure detection.

Despite these advantages, achieving robust underwater SLAM remains a formidable task due to the distinctive characteristics of underwater environments, including turbidity, dim lighting, and weak textures. These factors pose challenges for SLAM methods based on hand-crafted features such as SIFT [5], ORB [6], and Shi-Tomasi [7] in terms of ensuring consistent feature extraction and tracking. To address these issues, methods of image enhancement on image frames before feature extraction were proposed by scholars [8,9]. For example, an adversarial contrast learning method in [8] was designed for addressing underwater image degradation in visual SLAM. Considering the issue of water turbidity, ref. [9] proposed a model and model-free hybrid image enhancement approach. While the enhancement feature extraction works to some extent, challenges persist in obtaining accurate keypoint detection and corresponding descriptors due to the weak texture characteristics of underwater scenes.

On the other hand, scholars have tried to incorporate deep features into SLAM [10,11,12]. Methods based on deep learning possess the capability to process full-size images and simultaneously compute pixel-level keypoints and their descriptors. In GCN-SLAM [10], GCNv2 is integrated into ORB-SLAM2 [13] to predict the location keypoints and descriptions in images. HF-NET [14] is incorporated into ORB-SLAM2, resulting in a robust and efficient DXSLAM system [11]. To enhance the system stability, ref. [12] introduced a novel deep learning SLAM system that utilizes self-supervised learning to optimize the network. While deep learning-based SLAM methods often outperform traditional approaches in complex environments, they frequently require substantial labeled datasets for training. However, issues such as degradation of underwater images, such as blue-green bias and blurring, can affect the precise definition and labeling of keypoints.

Therefore, this paper presents a visual SLAM method named RU-SLAM that is subjected to dim and weakly textured underwater environments. It specifically addresses issues of weak texture, image degradation, and the difficulty in accurately labeling keypoints. In this system, an underwater image feature extraction network (UWNet) is integrated to extract features from input images. UWNet incorporates a channel attention [15] and a position attention module [16] into the local keypoint extraction branch. Additionally, it integrates a deformable convolutional module [17] into the local descriptor generation branch to enhance the accuracy of local keypoints and descriptors. To address the challenge of inaccurate keypoint labeling, a pseudo underwater image generator is designed based on a physical model, and UWNet is trained using a self-supervised approach. In summary, the contributions of this work are as follows:

1. A visual SLAM system, based on a deep feature extractor, demonstrates robust functionality in dimly lit and weakly textured underwater environments. This system efficiently extracts both local and global features from underwater images, facilitating tracking and loop closure detection threads, respectively.
2. A pseudo-underwater image generation method, based on a physical model, accurately simulates real underwater scenes. Utilizing this method as an intermediary, UWNet undergoes training via self-supervised learning. This process involves employing a teacher model to extract information from aerial images and guide UWNet’s learning process, enabling it to acquire the necessary knowledge effectively.
3. The experiments conducted on the public EuRoC land dataset [18], the AQUALOC underwater public dataset [19], and our Pool dataset showcase the exceptional localization performance and robustness of RU-SLAM, especially in challenging conditions characterized by weak texture and low lighting in underwater environments.

The structure of this paper is as follows: Section 2 provides a concise overview of the relevant literature. Section 3 presents detailed enhancements made to RU-SLAM. Experimental results and analysis of the proposed method are presented in Section 4. Finally, Section 5 concludes the paper.

## 2. Related Works

### 2.1. Works Based on Deep Learning for Feature Extraction

The algorithm for extracting features based on deep learning can be broadly categorized as weakly supervised or self-supervised. Weakly supervised methods primarily focus on optimizing the network using actual image pose information. For instance, CAPS [20] leverages the camera pose to establish antipodal constraints between image frames, optimizing network weights and demonstrating enhanced performance on various geometric tasks. Based on CAPS, a decoupled training strategy is proposed that employs a line-to-window search approach utilizing image pose values to effectively narrow down the search space for enhanced descriptor learning [21]. However, applying such training methods in underwater scenarios poses challenges, particularly when the pose accuracy obtained by sensors falls short of training requirements.

In recent years, self-supervised learning approaches have gained more attention. SuperPoint [22] utilized self-supervised learning to simultaneously train the feature extraction and descriptor generation networks. LF-Net [23] employed attention and asymmetric gradient backpropagation mechanisms for separate self-supervised learning. R2D2 [24] proposed a novel loss function based on micro-averageable accuracy for improving the reliability and repeatability of features, while ASLFeat [25] introduced deformable convolution. For computational efficiency, HF-NET [14] incorporated knowledge distillation, using [22,26] as teacher networks and designing a lightweight MobileNetV2-based [27] student network to extract image features. These advancements highlight that self-supervised learning methods can extract relatively accurate keypoints and descriptors without manual labeling. Therefore, a novel approach is proposed to tackle the issue of inaccurate image keypoint feature labels by employing self-supervised learning for both local and global feature extraction.

### 2.2. Visual SLAM

Traditional approaches of visual SLAM can be divided into direct and feature-based methods based on distinct data processing techniques. These methods, like DTAM [28], SVO [29], and LSD-SLAM [30], directly employ image brightness information to estimate the current location of the body. The other approach estimates the pose by extracting and matching keypoint features, where Shi-Tomasi [7] and ORB [6] are the more popular feature extraction algorithms. For instance, MonoSLAM [31], ORB-SLAM3 [32], and VINS-Mono [33] have extensively utilized them. The direct method, although efficient, is sensitive to changes in ambient lighting and is more challenging to initialize compared to the feature-based method, which exhibits poor performance on less textured or repetitive images.

Considering the challenges, visual SLAM based on deep learning has gradually emerged as a prominent research focus within the broader SLAM domain. These approaches typically employ deep learning networks to implement certain functionalities within traditional SLAM algorithms. For instance, GCNv2-SLAM [10] uses a graph neural network to generate image keypoint features. DXSLAM [11] leveraged a deep CNN, enhancing the stability of the SLAM system in intricate environments. RWT-SLAM [34] replaced the conventional SLAM module with a CNN-based LoFTR [35] network in ORB-SLAM2 to generate the description sub-module. The proposed study presents a cross-modal knowledge distillation framework, seamlessly integrated into the ORB-SLAM3 architecture [36]. In summary, deep learning-based methods outperform traditional visual SLAM in complex environments. However, these approaches have not effectively addressed challenges in underwater scenarios, such as weak texture, image degradation, and inaccurate keypoint labeling. Consequently, this paper introduces an underwater visual SLAM method based on deep learning, RU-SLAM.

## 3. RU-SLAM Method

### 3.1. Overview

**Figure 1:**
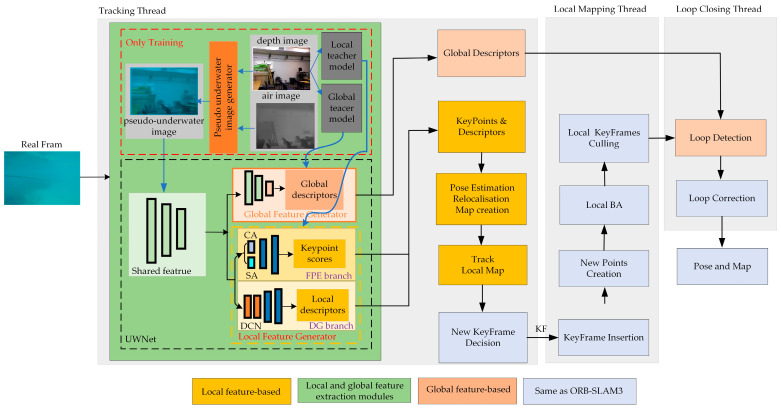
The overall framework of the RU-SLAM system.

The overall framework of the RU-SLAM is illustrated in Figure 1. This system is constructed upon ORB-SLAM3 with the underwater image frame (Real Frame) serving as input. It can be broadly categorized into three main parts based on thread tasks: tracking, local mapping, and closed-loop detection.

In the tracking thread, UWNet acts as an extractor to obtain local and global features of Real Frame. The channel attention (CA) and spatial attention modules (SA) are added at the forefront of the local feature point extraction branch (FPE) to process the input features in parallel before fusing the features. Furthermore, we introduce two deformable convolution (DCN) modules at the descriptor generation branch (DG). In the training phase, both the in-air RGB image (air image) and the pseudo-underwater image are utilized as inputs for the teacher models and the student mode, respectively. The information extracted from these teacher models is utilized to train the student model.

Local keypoint features and descriptors play a crucial role in system initialization, camera pose estimation, and determining keyframes (KFs) and their insertion into the local mapping thread. Upon receiving new KFs, the local mapping thread updates KFs and their connection relationships within the local map. Simultaneously, Bundle Adjustment (BA) is performed to optimize the position and map points of KFs in the current local map. Redundant KFs and their corresponding map points are then eliminated. The possibility of loop closure is assessed using a similarity score, which is computed based on the global descriptors of both the current KF and the KFs stored in the database. Geometric verification and temporal geometry verification are subsequently employed to determine whether correction for loop closure is necessary in the present region. Specific details of the improvement module are elaborated below.

### 3.2. Local and Global Feature Extraction Module

**Figure 2:**
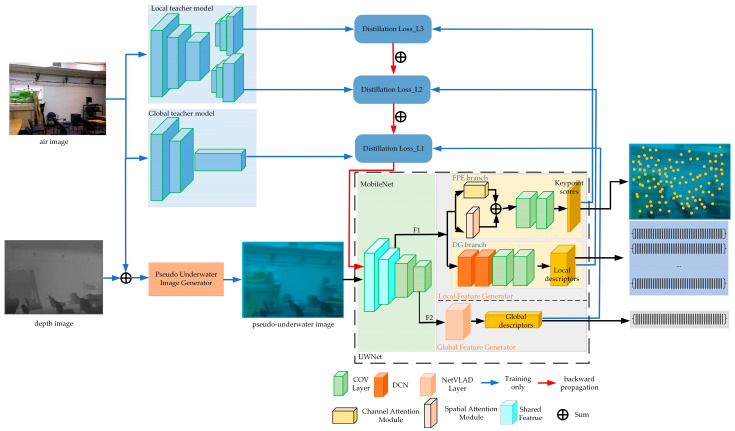
Framework of local and global feature extraction methods for images.

The approach for local and global feature extraction, based on self-supervised learning, is depicted in Figure 2. Initially, the proposed pseudo-underwater image generator is employed to acquire simulated underwater images (pseudo-underwater images). Subsequently, both local teacher models, SuperPoint, and global ones, NetVLAD, are utilized for feature extraction from the air image to guide UWNet in extracting corresponding features from the pseudo-underwater image. This process effectively mitigates the challenges associated with underwater image degradation. In the subsequent sections, the pseudo-underwater image generator and the UWNet components are described in more detail.

#### 3.2.1. Pseudo Underwater Image Generator

1 Improved underwater imaging model

A widely employed classical physical model for underwater imaging is the Jaffe–McGlamery model [37]. Reputable scholars have observed that the limited visible range in underwater optical imaging often results in the general exclusion of the foreground scattering component.

The expression is presented in Equation (1).

(1) $$M^{\lambda }(x)=N^{\lambda }(x)e^{-\beta (\lambda )d(x)}+A^{\lambda }(1-e^{-\beta (\lambda )d(x)}),\lambda \in \{r,g,b\}$$

(2) $$t^{\lambda }(x)=e^{-\beta (\lambda )d(x)}$$

where *x* represents the pixel coordinate value, λ stands for the color channel, $M^{\lambda }(x)$ denotes the synthesized underwater image, $N^{\lambda }(x)$ represents the RGB image in air, $A^{\lambda }(x)$ signifies the global background light, $t^{\lambda }(x)$ denotes optical medium transmittance, β(λ) is the attenuation coefficient of the water body, and d(x) is the distance from the target scene to the imaging device scene depth. The components of $N^{\lambda }(x)e^{-\beta (\lambda )d(x)}$ and $A^{\lambda }(1-e^{-\beta (\lambda )d(x)})$ are respectively responsible for direct attenuation and background light scattering. Additionally, attenuated atmospheric light and the auxiliary light sources are considered in underwater scenes. Equation (2) can be refined to Equation (3) following the approach suggested by [38].

(3) $$\begin{array}{ll} M^{\lambda }(x) & =A^{\lambda }R^{\lambda }\left(x\right)t^{\lambda }(x)+A^{\lambda }(1-t^{\lambda }(x))\\ & =L(x)\eta^{\lambda }(x)R^{\lambda }(x)t^{\lambda }(x)+L(x)\eta^{\lambda }(x)(1-t^{\lambda }(x)) \end{array}$$

In this Equation (3), $N^{\lambda }=A^{\lambda }R^{\lambda }(x)$, $A^{\lambda }=L^{\lambda }(x)\eta^{\lambda }(x)$, $R^{\lambda }(x)$ represents the reflected light, L(x) is the illumination intensity, and $\eta^{\lambda }(x)$ is the ambient light color.

The proposed model considers various factors to create a more realistic underwater environment. To simulate particulate impurities in the underwater images, Gaussian noise W is added to the model. Furthermore, image blurring may occur due to the movement of the AUV. Through the convolution operation, both the point spread function (PSF) and the direct transmission attenuation component are used to replicate image blurring. Overall, the modified model as Equation (4).

(4) $$M^{\lambda }(x)=\{L(x)\eta^{\lambda }(x)R^{\lambda }(x)e^{-\beta (\lambda )d(x)}\}*PSF+L(x)\eta^{\lambda }(x)(1-e^{-\beta (\lambda )d(x)})+W$$

2 Estimation of $A^{\lambda }$ and $t^{\lambda }$

In general, the ambient illumination varies gently throughout the space except for the occluded region. Therefore, this paper assumes that $A^{\lambda }$ is constant in any localized region donate $\Omega_{i}$. The value *V* of Equation (3) in the $\Omega_{i}$ can be obtained by Equation (5).

(5) $$V=\underset{x\in \Omega_{i}}{\max}M^{\lambda }(x)=\underset{j\in \Omega_{i}}{\max}(L_{\Omega_{i}}(x)\eta_{\Omega_{i}}^{\lambda }(x)R^{\lambda }(x)t_{\Omega_{i}}^{\lambda }(x)+L_{\Omega_{i}}(x)\eta_{\Omega_{i}}^{\lambda }(x)(1-t_{\Omega_{i}}^{\lambda }(x)))$$

The transmittance $t_{\Omega_{i}}^{\lambda }(x)$ is locally invariant from [39]. According to the priori theory of maximum reflection proposed in [38], it can be obtained that $\underset{x\in \Omega_{i}}{\max}R^{\lambda }(x)\approx 1$. Thus Equation (6) can be deduced.

(6) $$\eta_{\Omega_{i}}^{\lambda }(x)=\frac{V}{L_{\Omega_{i}}(x)}$$

based on the above assumption of in-variance in the local region, we can obtain Equation (7).

(7) $$L_{\Omega_{i}}(x)=\underset{x\in \{R,G,B\}}{\max}(\underset{x\in \Omega_{i}}{\max}V)$$

The value of $t^{\lambda }$ is related to β(λ) and d(x) according to Equation (2). Since the dataset used for synthesis includes depth data, there is no need for the estimation of d(x). We employed ten water types proposed by Jerlov [40] to estimate β(λ).

#### 3.2.2. UWNet Method

1 Network architecture

The degradation of underwater images causes extracting local and global features to be a complex task. We devised the UWNet network based on the inspiration from HF-Net, as shown in Figure 2. It utilizes MobileNetV2 as a backbone network to extract high-level features from the input image. The first seven layers of this backbone network serve as shared features, producing a feature map F1 at 1/8 resolution in the seventh layer and a feature map F2 at 1/32 resolution in the eighteenth layer. Subsequently, F1 and F2 are respectively used as inputs for both the local and global feature generators.

In the FPE branch, the feature map F1 is first inputted into both the SA and the CA to capture dependencies across spatial and channel dimensions. Their outputs from SA and CA are then fused to enhance the feature representation. Subsequently, the fused features pass through two consecutive convolutional blocks. The first block involves a convolution operation, followed by batch normalization (BatchNorm) and ReLU activation. This is followed by another convolutional operation with a 1 × 1 kernel size, resulting in 65 channels. The resulting features are reshaped using a SoftMax operation to generate the keypoint score map.

Concurrently, the feature map F1 is processed in the DG branch, where two deformable convolutional layers are utilized to capture crucial feature information and improve learning of scale kernel and rotation transformations. Following this, two convolutional operations are performed. The first convolutional block includes a 3 × 3 convolutional operation, BatchNorm, and ReLU activation. In contrast, the second convolutional block consists solely of a 1 × 1 convolutional operation with 256 channels. Finally, the output features undergo bilinear interpolation and L2-Norm operation to generate the local feature description subgraph.

The global feature generator is utilized to acquire the image’s global descriptor. F2 is fed into the NetVLAD layer and then undergoes a dimensionality reduction operation to obtain a global descriptor of size (4096). NetVLAD is primarily used for re-recognition or location tasks.

2 Train details

We employ the concept of knowledge distillation to optimize UWNet network parameters in a self-supervised multitasking manner, utilizing the RMSProp optimizer with an initial learning rate set to 0.003, employing a batch size of 16, and conducting training for 150 epochs. Traditional multitask learning typically involves jointly optimizing all losses and manually adjusting their weights. However, this approach often struggles to produce models that excel simultaneously across multiple tasks. Following the training strategy outlined in [14], the automatic selection of weights for different losses in multi-task learning and self-adjustment during training have been proven effective in enhancing the accuracy of individual tasks. Hence, we utilized Equation (8) as the loss function.

(8) $$L=e^{-\alpha }L_{1}+e^{-\beta }L_{2}+2e^{-\gamma }L_{3}+\alpha +\beta +\gamma$$

where $L_{1}={\left\|D_{s}^{g}-D_{t_{1}}^{g}\right\|}_{2}^{2}$, $L_{2}={\left\|D_{s}^{l}-D_{t_{2}}^{l}\right\|}_{2}^{2}$, and $L_{3}=CE(P_{s},P_{t_{2}})$. The values of α, β and γ are obtained by learning through network training. Furthermore, *t*_1_, *t*_2_, and *s* are respectively the NetVLAD, SuperPoint, and UWNet network. The global and local descriptors are $D^{g}$ and $D^{l}$, respectively.

### 3.3. Feature Tracking

The tracking thread utilizes local keypoint features and descriptors extracted by UWNet in each input image frame to estimate camera motion. To be specific, the system was initially initialized. Subsequently, the keypoint features from the previous frame were projected onto the current frame at a constant speed, and matching was performed within a local window. In case of failure, an attempt was made to match between the current frame and reference KF. The standard k-nearest neighbor search and subsequent ratio test were implemented in our system for these two matching strategies. Afterwards, tracking of a reconstructed local map took place along with pose optimization. Finally, it was determined whether the current frame satisfies the criteria for being designated as a KF.

### 3.4. Loop Closing Module

Loop closure is crucial for eliminating cumulative errors in SLAM and establishing a globally consistent map. The current methodologies predominantly rely on the bag of words approach. However, this method neglects spatial relationships between features, potentially leading to false closures. Therefore, a closure detection method based on global descriptors has been employed in our system, which calculates the similarity between images based on global feature descriptors to determine whether the AUV revisits a location. By retrieving KFs with higher scores from the global descriptor database as candidate loops, the system mitigates the risk of false closures. Similarity judgment is performed using Euclidean distance calculation, as presented in Equation (9).

(9) $$score(x,y)=1-\sqrt{\sum_{i}^{N}{\left(v_{i}(x)-v_{i}(y)\right)}^{2}}$$

where v(x) and v(y) are the global feature vectors of the query image and the image in the database, respectively, *i* represents the elements of the vector, and the size of *N* is 4096.

## 4. Experimental Analysis

The authenticity of the simulated underwater images is first validated, followed by an assessment of UWNet’s accuracy as a local feature generator compared to other algorithms. Finally, diverse datasets are used to evaluate the complete RU-SLAM system. The experiments are conducted on a computer equipped with an Intel Core i9-9900K CPU (3.60 GHz × 16 cores), 16 GB of RAM, and an NVIDIA RTX 2080Ti GPU.

### 4.1. Experiments with Pseudo-Underwater Image Generator

**Figure 3:**
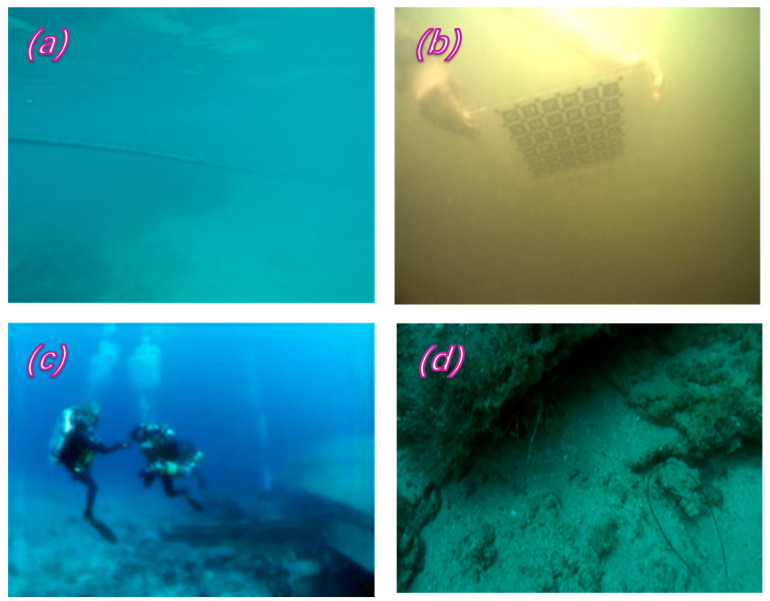
Underwater real image. (**a**) blue-green (**b**) turbidity (**c**) bluish (**d**) greenish.

A dataset comprising N images with varying degrees of degradation and low texture was selected from the EUVP dataset [41] to estimate the parameter value $A^{\lambda }$, as illustrated in Figure 3. Additionally, 200 images were randomly chosen as a test dataset from the NYU depth V2 dataset [42], which encompasses a total of 1449 RGB-D images. The specific details are as follows.

**Figure 4:**
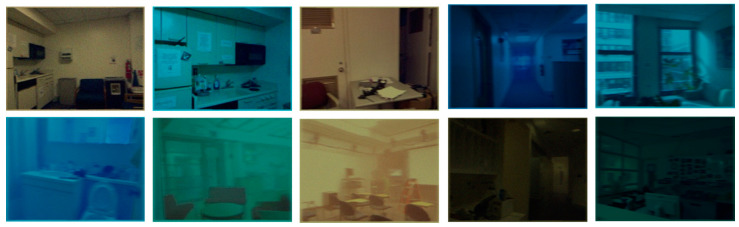
Pseudo-underwater images generated with different attenuation coefficients.

P regions of size 8 × 8 are randomly selected for each real underwater image. Equations (6) and (7) are then employed to calculate $A^{\lambda }$ within each region, resulting in $A^{\lambda }$ of N∗P denoted as $A^{*}$. In this paper, P and N are set to 8 and 10, respectively. For each image to be synthesized, one region is randomly selected from $A^{*}$ as $A^{\lambda }$, and $t^{\lambda }$ is obtained using Equation (2). The blur angle and length of the PSF function is 20 degrees and 10 pixels, while W is random Gaussian noise. The partially synthesized image data depicted in Figure 4 shows that the pseudo-underwater image exhibits varying background illumination, along with randomly added noise and different degrees of blurring. The clarity and contrast of the image progressively deteriorate under different attenuation coefficients, resembling the visual effect of real underwater images.

We employ a reference-free metric to evaluate the authenticity of pseudo-underwater images; it comprises the Underwater Color Image Quality Evaluation (UCIQE), Underwater Image Quality Measurement (UIQM), and its two attributes: Underwater Image Colorimetric Measurement (UICM) and Underwater Image Sharpness Measurement (UISM). The experimental results, as presented in Table 1, indicate that our algorithm can synthesize a more realistic underwater image compared to algorithms such as UWCNN [43], WaterGAN [44], and UGAN-P [45] for underwater image synthesis.

### 4.2. Evaluation of Local Keypoint and Descriptor in UWNet

The performance of the proposed local keypoint and descriptor in UWNet is assessed based on keypoint repeatability (%Rep.), matching score (%M.S.), and average matching accuracy (%MMA). Two datasets are utilized for evaluation: the Hpatches dataset [46] and a self-collected Underwater dataset (Upatches). The Upatches dataset comprises 30 original images obtained from the UIEB underwater public dataset [47], along with their corresponding homography-transformed images. These images exhibit characteristics of low-light conditions, turbidity, and low-texture underwater scenarios. It is noteworthy that the rotation angle does not exceed 180°.

The experiments were conducted using a dataset resolution of 480 × 640 and a limited number of feature points (2 k). According to the results presented in Table 2 on the Hpatches, our algorithm demonstrates competitive performance by outperforming other algorithms in terms of %M.S. and %MMA. For dataset Upatches, the proposed algorithm consistently outperforms other algorithms across all metrics. The experimental results based on these datasets reveals that our algorithm evinces commendable performance in both terrestrial and underwater datasets. Notably, the comparison of our algorithm’s performance with and without incorporating attention modules and deformable convolutions (Our w/o AM DCN) demonstrates a significant improvement in matching accuracy, thereby highlighting the efficacy of the proposed approach.

**Figure 5:**
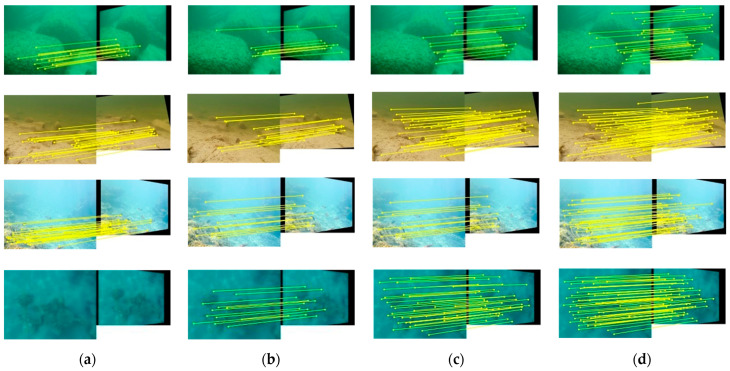
Results of image keypoint matching using different methods on the Upatches dataset: (**a**) SIFT; (**b**) LF-NET; (**c**) HF-NET; (**d**) Ours.

The visual results depicted in Figure 5 showcase the keypoint matching outcomes in selected Upatches data, where the yellow line represents correct correspondences. Even after RANSAC processing, our method consistently yields denser and more accurate matches compared with SIFT, LF-NET, and HF-NET, and this is especially evident in underwater images characterized by weak textures, low contrast, and blurring.

### 4.3. SLAM System Evaluation

The performance of RU-SLAM in both underwater and land environments is assessed using publicly available datasets, namely EuRoC and AQUALOC, as well as our own collected dataset called Pool. The Root Mean Square Error (RMSE) of the Absolute Pose Error (APE) and Relative Pose Error (RPE) between the estimated trajectory and the provided ground truth trajectory is computed using the evo tool as performance evaluation metrics, providing assessments of both global and local accuracy of the system. It should be noted that during the evaluation analysis, alignment is performed between the experimentally estimated trajectory and the provided ground truth. Specifically, in stereo vision scenarios, SE (3) transformation is employed for alignment, while in pure monocular vision, Sim (3) transformation is used to correct for scale due to a lack of depth information. Additionally, a relative interval of 0.1 m is set for RPE to enable more precise detection of positional changes and a thorough analysis of system performance. If the estimated trajectory is too short or diverges, the results will be marked as a failure (×). Furthermore, the average (Avg) in the table represents the mean of the sequences wherein all algorithms employed in the table achieve success.

#### 4.3.1. EuRoC Dataset

The EuRoC dataset comprises two sections, namely Machine Hall and Vicon Room, which include stereo image and IMU data. The first dataset consists of five sequences: MH01, MH02, MH03, MH04, and MH05. These sequences are categorized into simple, medium, and difficult levels based on lighting conditions and scene textures. The Machine Hall dataset is employed for conducting experiments on stereo visual SLAM. The robustness of RU-SLAM is evaluated through a comparative analysis with ORB-SLAM3 and DXSLAM, based on results as presented in Table 3.

Where ORB-SLAM3 is the latest visual SLAM based on the ORB, and DXSLAM integrates the feature extractor based on the CNN deep convolutional network into the ORB-SLAM2 framework, the RU-SLAM exhibits superior performance compared to other methods in four sequences of the dataset, as evidenced by the observations in Table 3. Compared to ORB-SLAM3, our algorithm achieves a reduction in error of 44.45% and 49.15% on the MH01 and MH04 sequences, respectively. This success is primarily attributed to the simplicity of MH01, featuring a well-lit and textured scene conducive to obtaining more uniform feature points through the deep learning-based method. Conversely, MH04, being a challenging sequence with a large viewing angle and low light, exposes the limitations of traditional SLAM based on visual feature points in such conditions. The trajectory errors in sequences MH02, MH03, and MU04 are further reduced by our method compared to the ORB-SLAM3. However, the extent of error reduction in MH02 is not as significant as that observed in DXSLAM. The above analysis reveals that our algorithm displays admirable performance in land scenarios characterized by wide viewing angles and low lighting conditions.

#### 4.3.2. AQUALOC Dataset

The AQUALOC dataset provides monocular, IMU, and depth data for underwater environments. It is categorized into two sections: harbor and archaeological, based on distinct acquisition environments. Additionally, it includes a ground truth trajectory that exhibits relatively high accuracy. To evaluate the performance of RU-SLAM in underwater scenarios, separate experiments were conducted using these two datasets of monocular data.

1 Archaeological

The archaeological dataset consists of 10 sequences collected on the seafloor at depths of several hundred meters. However, the propellers of the remotely operated vehicle (ROV) caused sediment disturbance on the seafloor, thereby introducing disruptions to visual feature recognition.

**Figure 6:**
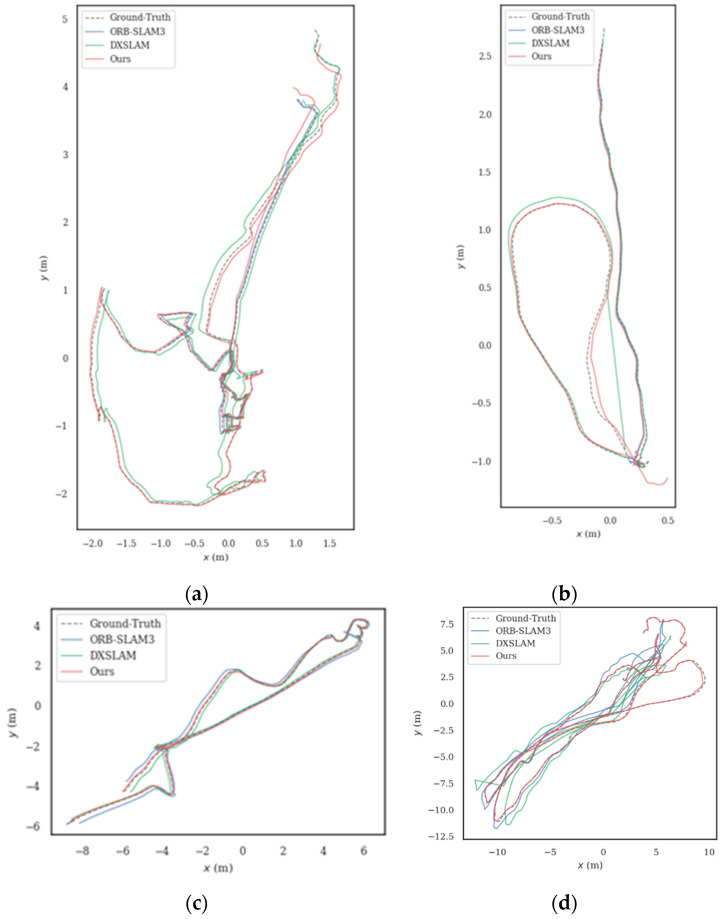
Comparison of partial sequence trajectory results from different algorithms in Archaeological datasets. (**a**) Sequences 01; (**b**) Sequences 03; (**c**) Sequences 05; (**d**) Sequences 07.

Detailed comparison results for the RMSE of ORB-SLAM3, DXSLAM, and RU-SLAM under APE and RPE are provided in Table 4. Additionally, Figure 6 presents trajectory comparisons across different algorithms for selected sequences. The superiority of the proposed method over others is evident from Table 4. Owing to interference from seafloor sediment and a lack of sufficient seafloor texture features, ORB-SLAM3 exhibits poor performance in this dataset. The average APE and RPE of the RU-SLAM algorithm decreased by 69.95% and 55.50%, respectively, in contrast to DXSLAM, thereby demonstrating the enhanced accuracy and robustness of our method.

2 Harbor

The harbor dataset comprises a total of seven sequences capturing scenarios of coastal ports at depths of 3–4 m. In this experiment, the study primarily focuses on the first six sequences, and present comparative test results between the ORB-SLAM3 and HFNET-SLAM algorithms and the proposed RU-SLAM algorithm. HFNET-SLAM refers to an algorithm where the unimproved HF-NET is used instead of the feature extraction module in ORB-SLAM3.

According to Table 5, our algorithm exhibits superior performance in underwater environments compared to the other two methods. However, the APE differences among the three algorithms in sequence 03 are minimal. This can be attributed to the clear water quality and abundant texture of this sequence, which facilitate both deep learning and traditional methods, enabling the attainment of satisfactory experimental results. In Sequence 04, both ORB-SLAM3 and HFNET-SLAM encounter localization failures due to the presence of visually poor features, resulting in incomplete positioning trajectories. In contrast, RU-SLAM successfully extracts features and accurately estimates camera poses in this challenging visual environment. Additionally, experimental results demonstrate that RU-SLAM exhibits enhanced robustness and stability compared to the original HFNET-SLAM.

#### 4.3.3. Pool Dataset

**Figure 7:**
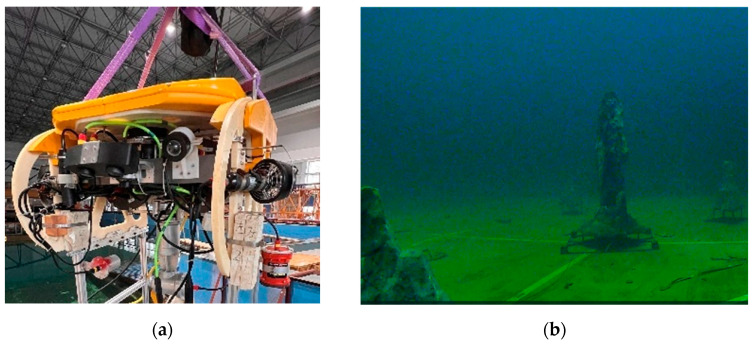
The ROV and Pool environment, including (**a**) Physical drawing of ROV; (**b**) Pool Rockery Placement.

In addition to utilizing the publicly available data set, we conducted our own data collection to assess the performance of RU-SLAM. Three sequences are captured by an ROV, as shown in Figure 7a, equipped with a monocular camera in a 30 × 50 × 10 m^3^ pool characterized by low illumination and weak textures. It is worth noting that the velocity of the ROV ranges between 0.3 and 0.5 m/s. There are artificial rock formations of various sizes strategically positioned at the bottom to function as obstacles. The ROV was remotely controlled via a console to navigate around obstacles and its movements were recorded during the experiment. The actual setup is illustrated in Figure 7b. The sequences consisted of the following: 01 circumnavigating the four sides of the pool with a maximum turn angle of 90 degrees; 02 maneuvering around obstacles within the pool in a complete circle; and 03 circumnavigating the large obstacle at the center of the tank bottom and forming a closed loop.

**Figure 8:**
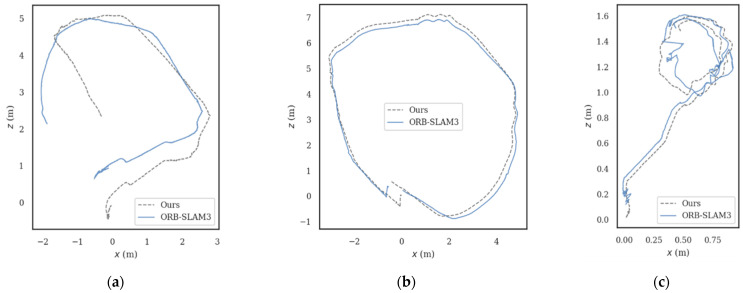
Estimated trajectories resulting from RU-SLAM and ORB-SLAM3 in our own Pool dataset. (**a**) Sequence 01; (**b**) Sequence 02; (**c**) Sequence 03.

The estimated trajectory results, obtained by comparing our system with ORB-SLAM3, are depicted in Figure 8. It is noteworthy that this component serves as a valuable complement due to the absence of ground truth measurements. As depicted in Figure 8a, when the texture at the bottom and walls of the pool is weak and repetitive, ORB-SLAM3 provides an incorrect rotation prediction, resulting in the trajectory deviating from the actual walking path. In Sequence 02, both ORB-SLAM3 and our algorithm exhibit good localization performance. This is mainly because Sequence b was recorded in water five to six meters from the pool bottom and moved around artificial hills under optimal lighting conditions. As shown in Figure 8c, RU-SLAM demonstrates robust performance in handling images with weak texture.

## 5. Conclusions

A robust visual SLAM system based on depth features is proposed for accurate positioning in challenging underwater environments with low illumination and weak texture. We introduce the UWNet network to generate dense local and global features. Thanks to the diverse receptive field facilitated by the attention mechanism and deformable convolution, high quality descriptors for scenes with limited texture and image degradation can be obtained. A pseudo-underwater image generator is presented, and self-supervised training is employed for UWNet to make the extract features more adaptable to underwater scenes. Moreover, we seamlessly integrate UWNet into ORB-SLAM3, and our experimental results on diverse public datasets as well as our data demonstrate the exceptional accuracy and robustness of our system.

## Figures and Tables

**Table 1:** Evaluation of reference-free underwater image quality metrics on the same test set using different image synthesis methods. The best result is shown in the bold numbers.

Method	UCIQE	UIQM	UICM	UISM
WaterGAN	0.430	0.514	10.351	1.153
UGAN-P	0.381	0.413	8.490	1.004
UWCNN	0.328	0.234	8.183	**0.453**
Ours	**0.271**	**0.190**	**4.809**	0.652

**Table 2:** Quantitative evaluation results on Hpatches and Upatches datasets for image matching. The best result is shown in the bold numbers.

Method	Hpathes			Upatches		
	%Rep.	%M.S.	%MMA	%Rep.	%M.S.	%MMA
SIFT	35.80	28.10	65.40	35.99	29.90	70.86
LF-NET	42.44	32.27	62.60	28.68	17.69	30.00
SuperPoint	52.35	45.88	72.11	42.00	46.93	70.00
ASLFeat	**62.24**	45.10	72.28	52.65	34.76	58.64
HF-NET	54.80	45.32	72.50	55.91	46.49	63.33
Our *w*/*o* AM DCN	48.20	38.60	65.90	52.71	41.53	53.33
Ours	56.00	**47.00**	**74.40**	**57.00**	**47.28**	**73.33**

**Table 3:** The RMS of APE (m) obtained from various SLAM methods applied in the Machine Hall. The best result is shown in the bold numbers.

Sequence	ORB-SLAM3	DXSLAM	Ours
MH01	0.027	0.022	**0.015**
MH02	0.037	**0.015**	0.016
MH03	0.028	0.025	**0.022**
MH04	0.118	0.121	**0.060**
MH05	0.072	0.064	**0.043**
Avg	0.056	0.044	**0.031**

**Table 4:** The results are obtained from various SLAM methods applied in the archaeological dataset. The best result is shown in the bold numbers.

Sequence	ORB-SLAM3		DXSLAM		Ours	
	APE	RPE	APE	RPE	APE	RPE
01	× ^1^	×	0.108	0.017	**0.065**	**0.009**
02	×	×	×	×	×	×
03	×	×	0.154	0.095	**0.124**	**0.055**
04	1.450	0.507	2.931	0.492	**0.763**	**0.315**
05	0.263	0.130	0.135	0.039	**0.106**	**0.047**
06	×	×	×	×	**0.183**	**0.020**
07	1.468	0.219	0.891	0.197	**0.132**	**0.086**
08	**0.101**	0.049	0.153	0.660	0.114	**0.048**
09	×	×	0.036	**0.227**	**0.027**	0.282
10	0.343	0.031	0.550	0.018	**0.161**	**0.012**
Avg	0.725	0.187	0.932	0.281	**0.255**	**0.101**

^1^ Represents trace failure.

**Table 5:** The results obtained from various SLAM methods applied in the harbor. The best result is shown in the bold numbers.

Sequence	ORB-SLAM3		HFNET-SLAM		Ours	
	APE	RPE	APE	RPE	APE	RPE
01	0.172	0.033	0.108	0.022	**0.080**	**0.018**
02	0.932	0.430	0.472	0.418	**0.349**	**0.374**
03	0.030	0.022	**0.026**	0.009	0.028	**0.008**
04	×	×	×	×	**1.440**	**0.873**
05	0.141	0.054	0.093	0.043	**0.074**	0.041
06	0.040	0.020	0.026	0.014	**0.020**	**0.013**
Avg	0.263	0.112	0.145	0.101	**0.110**	**0.090**

## Data Availability

All the data that support the findings in this study are given by contacting the corresponding author.
